# Mercury (II) Removal From Stock Solutions Using Pulsed Electrochemical Technique Coupled With Biochar Adsorbent

**DOI:** 10.1002/open.202500395

**Published:** 2026-05-07

**Authors:** Werkne Sorsa Muleta, Lelisa Regea Mengistu, Madhappan Santhamoorthy, Perumal Asaithambi

**Affiliations:** ^1^ School of Chemical Engineering Jimma Institute of Technology Jimma University Jimma Ethiopia; ^2^ Faculty of Civil and Environmental Engineering Jimma Institute of Technology Jimma University Jimma Ethiopia; ^3^ School of Chemical Engineering Yeungnam University Gyeongsan Republic of Korea; ^4^ Department of Biotechnology and Chemical Engineering School of Engineering Faculty of Science, Technology and Architure Manipal University Jaipur Jaipur India

**Keywords:** biochar adsorbent, environmental sustainability, heavy metal, pulsed electro‐oxidation, wastewater treatment

## Abstract

Mercury (Hg) in water bodies is a major environmental concern due to its high toxicity and bioaccumulation in the food chain. Conventional treatment methods have limited effectiveness in removing mercury. This study explores a combined approach involving pulsed electrooxidation (PEO) and biochar adsorption for enhanced Hg removal from synthetic water. Biochar, produced from agricultural waste, was optimized for efficient oxidation. Batch adsorption experiments evaluated the performance of the biochar adsorbent, analyzing factors such as dosage, contact time, and initial Hg concentration. The integration of PEO and biochar adsorption resulted in a synergistic enhancement of Hg removal from synthetic water. For maximum Hg removal effectiveness, the PEO approach was applied, using electrode spacings of 0.5 and 1 cm. The process's conductivity was increased by adding sodium chloride. The results indicated optimal removal efficiencies of 97.37% and 96.24% with PEO alone and 98.92% and 97.899% when PEO was combined with magnetic biochar for 0.5 cm and 1 cm electrode spacing, respectively. At pH of 6.03, 34.15 of minutes of reaction time, 1.55 g/L of magnetic biochar, 0.43 amps of current, 0.45 Kwh/m^3^, electric energy consumed, and 227 mg/L of mercury concentration, these results were collected. According to the results, mercury (II) be effectively removed from aqueous solutions by using a combination of PEO and biochar adsorption.

## Graphical abstract

Pulsed electrooxidation combined with magnetic biochar adsorption achieved up to 98.9% Hg(II) removal from synthetic water. Optimal conditions (pH‐6.03, 34.15 min, 1.55 g/L biochar, 0.43 A, 227 mg/L Hg) enhanced efficiency beyond electrooxidation alone, demonstrating a synergistic, energy‐efficient treatment approach.

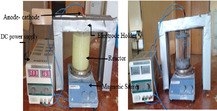

## 1 Introduction

All living things depend on water, which is also the most essential resource for human survival, after air. However, water resources can become contaminated through both natural processes and human activities [1]. Industrial, municipal, mining, agricultural, and other activities contribute to water pollution by releasing contaminants containing effluents [2]. These pollutants include heavy metals, pesticides, and polycyclic aromatic hydrocarbons, which pose risks to the environment and human health [3]. Industrial activities releasing chemicals, acids, bases, and hazardous heavy metals contaminate water [4]. Urbanization and industrialization have led to the generation of large volumes of untreated or inadequately treated wastewater, which often contains heavy metals that can cause cancer, organ damage, joint problems, and even death [5, 6].

Mercury (Hg) is of particular concern among heavy metals due to its high toxicity and persistence in the environment [7]. Industrial effluents are a major source of mercury contamination, as they often contain significant concentrations of heavy metals such as cadmium, copper, mercury, nickel, and lead [8, 9]. Mercury is a harmful contaminant that is found all over the world and is mostly caused by low‐level industrial sources [10]. It has an impact on fish populations, aquatic life, and human health. When water undergoes chemical and physical changes, such as alterations in color, odor, or chemical composition, it becomes wastewater [11]. The accumulation of heavy metals in the environment has become a pressing issue, as they can reach hazardous levels and pose risks to both biotic and abiotic systems [7]. The use of mercury in various industries has resulted in widespread environmental contamination [12, 13]

In the industrial manufacturing process, wastewater treatment is becoming a crucial component [14]. Various treatment methods have been proposed for the removal of heavy metals from aqueous waste streams [15]. These methods include biological, physical, chemical, advanced oxidation, and electrochemical techniques. However, the removal of heavy metals from wastewater can be challenging due to their non‐biodegradability and potential toxicity [16, 17] Precipitation, ion exchange, and adsorption are commonly employed techniques, but they often require considerable time, resources, and complex solutions [18].

EO is an efficient method for removing organic compounds from municipal wastewater, offering significant cost savings and reduced energy consumption compared to traditional microbial digestion methods [19]. The electrocoagulation process involves producing destabilizing agents such as Al and Fe, neutralizing electric charge, and bonding particles to form a mass, effectively removing pollutants from water, requiring no chemical use [20]. By dissolving an anode in water, metal ions are released, neutralizing suspended particles and creating bigger flocs [21]. Using an electric current, the process known as electro‐oxidation (EO) produces potent oxidizing agents in water that break down organic and inorganic contaminants into less dangerous forms [22]. It is employed in the degradation of hazardous substances, removal of persistent organic pollutants, disinfection of water, and treatment of industrial effluents [23]. The process of EO involves the direct oxidation of contaminants in water, generating reactive species like hydroxyl radicals or chlorine, which degrade organic pollutants, disinfect water, and treat wastewater [24].

Pulsed electrooxidation (PEO) occurs when an electric current is applied in a pulse rather than continuously [25]. Pulsing can prevent the passivation of electrodes, which is a common problem where layers of byproducts form on the electrode surface, reducing efficiency and enhancing mass transfer [26]. The process targets various contaminants, including organic compounds, heavy metals, and pathogens [25]. The radicals and other oxidizing agents break down these contaminants into less harmful substances such as carbon dioxide, water, and mineral salts [27]. It has emerged as an efficient and cost‐effective method for the treatment and remediation of heavy metal‐contaminated wastewater. PEO involves the application of pulsed direct current to induce oxidation reactions, resulting in the removal and conversion of contaminants into less harmful forms [28, 29]. This technology has shown promise in reducing pollution levels by eliminating trace metals and suspended particles [30]. It is particularly effective for the removal of mercury from wastewater, offering a practical and sustainable solution for its remediation [31].

PEO is a promising wastewater treatment method, but its scalability is crucial for large‐scale industrial and municipal applications [21]. Factors influencing scalability include system design and configuration, energy requirements, operational costs, treatment capacity, and integration with existing infrastructure [32]. Durable electrode material and modular reactor design can enhance efficiency and longevity. Energy efficiency and power supply are also essential. Integrating with existing infrastructure can facilitate scalability [33]. This procedure involves applying a pulsed electrical current to wastewater‐based electrodes, which creates reactive species capable of oxidizing mercury and changing it into less dangerous forms [31, 34]

To produce reactive species that can oxidize mercury into less hazardous forms, a pulsed electrical current is applied to electrodes. As indicated in the following reaction, mercury can oxidize, produce reactive oxygen species (ROS), be oxidized by hydroxyl radicals, and precipitate mercury compounds [35, 36]. Responses may vary depending on the specific conditions and the materials used for the electrodes [37, 38]. In the absence of biochar, pulsed EO results in the following reaction:

(1) $${\mathrm{Hg}}^{2+}+O^{•}\;\to \;\mathrm{HgO}$$

Oxygen evolution reaction:

(2) $$2H_{2}O\;\to \;4H^{+}+4e^{-}+O_{2}$$

Hydroxyl radical formation:

(3) $$H_{2}O\;\to \;{\mathrm{HO}}^{•}+H^{+}+e^{-}$$

Oxidation reaction:

(4) $${\mathrm{Hg}}^{2+}+\mathrm{OH}\;\to \;\text{Hg}{\mathrm{(OH)}}^{2}$$

Preciptation Hg compound [39, 40]:

(5) $$\mathrm{Hg}{\left(\mathrm{OH}\right)}_{2}\;\to \;\mathrm{HgO}+H_{2}O$$

Coupling pulsed electrochemical oxidation (PECO) and magnetic biochar can enhance mercury removal efficiency and scalability [41]. Magnetic biochar adsorbs contaminants, reduces electrode fouling, and reduces the load of PEO while PEO degrades them [42]. This method can achieve higher efficiency, lower operational costs, and greater adaptability to different wastewater scales and types. The process involves the formation ofROS, oxidation of mercury, and adsorption onto biochar, enhancing mercury removal efficiency [29].

Hydroxyl radical formation:

(6) $$H_{2}O\;\to \;{\mathrm{HO}}^{•}+H^{+}+e^{-}$$

Superoxide anion formation

(7) $$O_{2}+e^{-}\;\to \;O_{2}^{-}$$

Oxidation reaction with hydroxyl radical:

(8) $$\text{HgO}+\mathrm{OH}\;\to \;{\mathrm{Hg}}^{2+}+H_{2}O$$

Oxidation reaction with superoxide:

(9) $$\mathrm{HgO}+O_{2}\;\to \;\mathrm{HgO}+O_{2}^{-}$$

Adsorption:

(10) $$\text{Biochar}-\mathrm{OH}+{\mathrm{Hg}}^{2+}\;\to \;\text{Biochar}-O-{\mathrm{Hg}}^{+}+H^{+}(\mathrm{biochar}\;\mathrm{bound}\;\mathrm{Hg}\;\mathrm{complex})$$

Precipitation reaction:

(11) $${\mathrm{Hg}}^{2+}+\text{Biochar}\;\to \;\text{Biochar}-\mathrm{HgO}+H_{2}O$$

Reduction and adsorption [43]:

(12) $$\text{Biochar}-C+{\mathrm{Hg}}^{2+}\;\to \;\text{Biochar}-{\mathrm{Hg}}^{+}+{\mathrm{CO}}_{2}(\text{Biochar bounded elemental Hg})$$

During PECO, water electrolysis generates reactive oxygen species (ROS), primarily hydroxyl radicals (•OH) and superoxide radicals (O_2_•^−^). The highly oxidative •OH radicals rapidly attack Hg^2+^, converting it into Hg(OH)_2_, which subsequently dehydrates to form insoluble HgO, enabling its precipitation and removal from solution. Superoxide radicals, although less oxidative, help maintain an oxidative environment and support secondary oxidation reactions, stabilizing mercury in its oxidized form. When magnetic biochar is introduced, additional removal pathways are activated through surface complexation and adsorption via functional groups such as –OH and –COOH. The carbon matrix of biochar further enhances mercury immobilization and reduces electrode fouling. Overall, the integrated system combines oxidation, precipitation, and adsorption mechanisms, resulting in enhanced mercury removal efficiency and improved process stability.

Mercury‐contaminated wastewater poses significant environmental and health risks, as mercury is a highly flammable and poisonous heavy metal [43, 44]. Everyday discharges of heavy metal‐contaminated wastewater, whether direct or indirect, present a considerable risk due to the persistent nature of these pollutants in the environment [45]. The risks associated with heavy metal contamination, including mercury, have gained increased attention worldwide. Mercury exposure can lead to adverse effects on the nervous system, impaired renal function, hypertension, and impaired thyroid function [9].

In Ethiopia, for example, heavy metal contamination is a growing concern, causing significant harm to human health and the environment. PEO offers several advantages over other treatment methods [46]. Unlike traditional coagulation processes, PEO does not require the use of chemical coagulants [47]. Instead, the sacrificial anode, typically made of iron or aluminum, undergoes electro‐dissolution during the electrolysis process, generating the necessary coagulating agents. PEO has been successfully applied in the treatment of textile wastewater, demonstrating its effectiveness in removing heavy metals and agglomerating trace contaminants [48, 49].

The study focuses on enhancing the removal efficiency of mercury (II) from synthetic mercuric chloride wastewater by combining PEO with a biochar adsorbent. Biochar, derived from agricultural waste through pyrolysis, is known for its high adsorption capacity [50]. By synergistically employing pulsed electrochemical oxidation (PECO) and biochar adsorption, aim to reduce the negative effects of mercury contamination on the environment and increase the removal efficiency of mercury (II) ions [51]. The objectives of this research are to determine the optimal operating conditions for PECO, including pulse frequency, pulse duration, and current density, to achieve the highest removal efficiency of mercury (II) ions. Additionally, to evaluate the adsorption capacity of biochar for mercury (II) ions through batch adsorption experiments, considering factors such as contact time, initial concentration, and biochar dosage. This study contributes to the development of effective and sustainable technologies for the remediation of mercury‐contaminated wastewater. By elucidating the mechanisms involved in the combination of PECO and biochar adsorption processes, goal is to improve our comprehension of the removal mechanisms and offer suggestions for potential future uses. The findings of this study have implications for the preservation of the environment and the protection of human health.

## 2 Materials and Methods

### 2.1 Apparatus and Equipment

Specialized equipment of a variety of kinds, each chosen for its precision and dependability, was included in the experimental setting. Internal mixing in the reactor or electrochemical cell, where the treatment operations took place, was ensured by an Janke & Kunkel (JK) magnetic stirrer (model RHB2). Stable and controllable electrical power was provided for the electrochemical processes using an ANDELI DC power supply (type WYJ‐0‐15V/5A). An aluminum (Al) electrode was used in the arrangement to guarantee the best possible conductivity. To provide accurate pH monitoring, a pH meter (model pH 3310) from WTW was utilized, and a UV/Vis‐spectrophotometer r (model 6700) by JENWAY was utilized for concentration readings. Using a glass funnel from Pyrex to aid liquid transfer, the temperature was carefully regulated using a Fluke thermometer.

### 2.2 Chemicals Required

The experimental procedures involved the use of chemicals to prepare the synthetic wastewater and perform analyses. mercuric chloride (HgCl_2_), hydrochloric acid (HCl), sodium hydroxide (NaOH), sulfuric acid (H_2_SO_4_), and sodium chloride (NaCl). Distilled water was used as the solvent for preparing solutions. A 1000 mL mercury stock solution was prepared by dissolving mercuric chloride (HgCl_2_) in distilled water, sulfuric acid, and sodium hydroxide for pH Adjustments. All these chemicals and reagents were of analytical grade and were purchased from AE Chemicals Trading P.L.C (Addis Ababa, Ethiopia). Biochar adsorbent, derived from agricultural waste through pyrolysis, was used for batch adsorption experiments.

### 2.3 Experimental Setup

**FIGURE 1:**
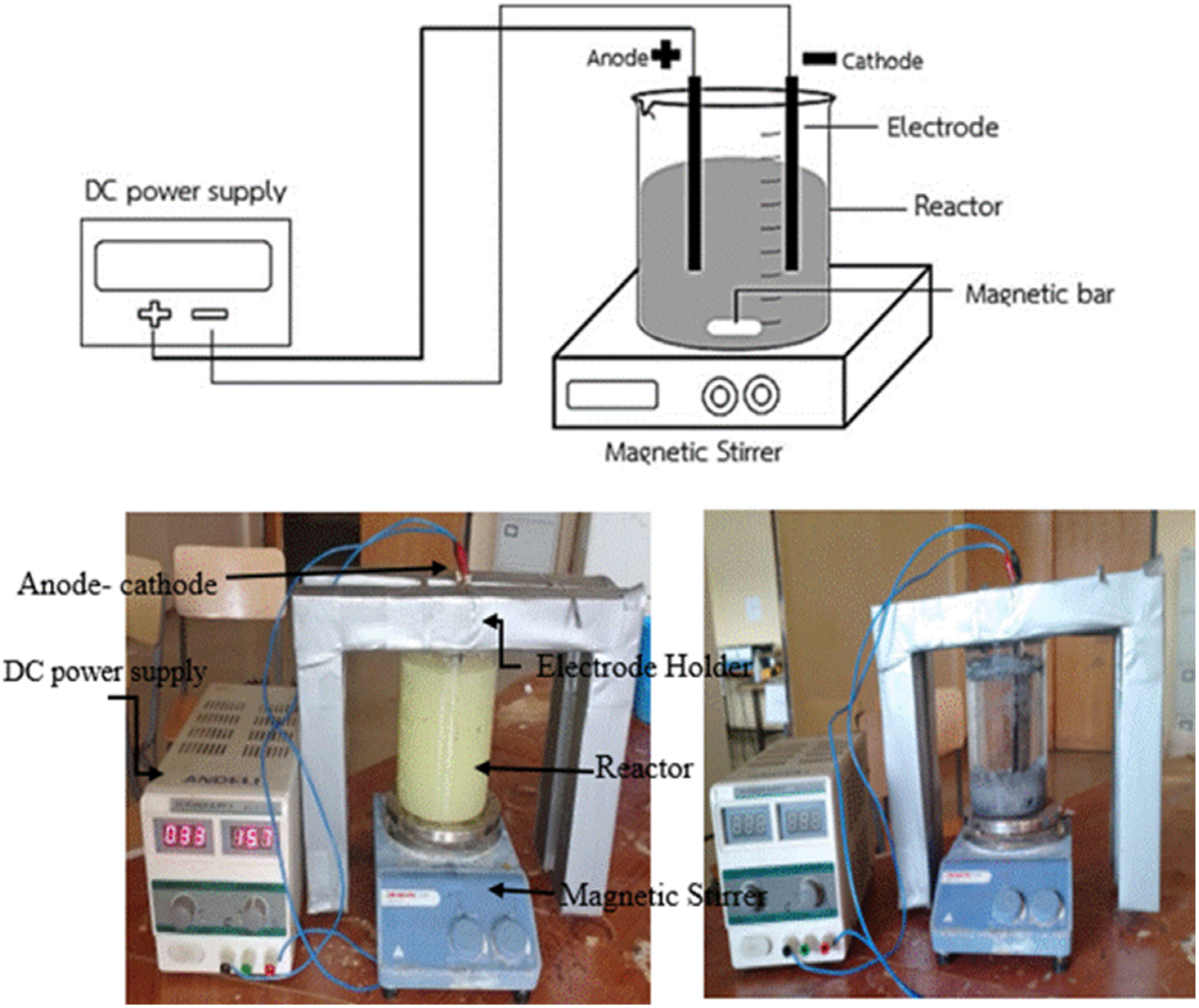
Pulsed Electrochemical assisted biochar Experimental setups.

The PEO process was conducted in a specially designed electrochemical cell to assess the performance of the PEO process for heavy metal‐contaminated wastewater treatment (synthetic wastewater treatment). As shown in Figure 1 [52], the process was carried out in a batch Plexiglas cylindrical reactor with flat, rectangular sheets of metal electrodes with dimensions of 15 cm (length) × 5 cm (width) × 1 mm (thickness). Subsequently, mercuric chloride stock solution was added. There was also a magnetic stirrer bar inside the beaker which contained the wastewater sample. The wastewater sample was placed in PEO cell along with an Al electrode that was connected to a cathode and an anode. There was a 0.5 and 1 cm measure performed between the electrodes. Next, electrical wires are used to link the anode and cathode electrodes to the DC power source. The elimination % of mercury (Hg) and power consumption were assessed by varying all operating parameters (pH, current, time, and electrode distance (ED)). In this experiment, electrodes made of Al were utilized.

PEO involves immersing electrodes in a mercury stock solution and exposing them to a periodic electric potential [53]. During the first part of the designated contact duration, the PEO reaction time, the first electrode is linked to the anode. For the second half of the reaction period, by exchanging the anode and cathode, the electrode is switched and linked to the cathode. Stirring was continuous to ensure that the oxidation process was distributed evenly throughout the entire duration. The mercury stock solution was typically subjected to direct current during the experiment, with regular changes of the cathode and anode based on the reaction time.

### 2.4 Sample Size

The most accurate representation for the treatment of synthetic wastewater was prepared by dissolving mercuric chloride in distilled water following lab scales. This chemical was prepared at various times. Furthermore, around 60 liters of synthetic wastewater were produced for the experiment depending on the number of experimental runs. The number of experimental runs determines the sample size (N) [49].

(13) $$N=k^{2}+2k+n=2^{n}+2n+c$$

Where *N* denotes the number of experimental runs, *n* is the number of repetitions (centers), and *k* is the number of distinct components included in the research. Finally, the central composite design (CCD) model is best explained by expert software design (Version 13.0). Based on this, the number of experimental runs is 60, which is equal to the sample size. There are 30 runs 0.5 cm ED and 30 for 1 cm distance. Finally, after treating the stock solution, the removal efficiency was estimated as follows:

(14) $$\%,\text{Mercury}\;\text{removal}=\left(\frac{Hg_{i-}Hg_{t}}{Hg_{i}}\right)*\left(100\right)$$

where Hg_*i*_ and Hg_*t*_ are concentrations of mercury before and after treatment, respectively

**Electric Energy Consumption (kWh/m^3^)** [54, 55]

(15) $$\text{Electric}\;\text{energy consumed}=\frac{VIt}{\text{Vs}},\left(\frac{\text{kWh}}{m^{3}}\right)$$

where *V* = voltage, *I* = current, *t* = time and Vs = volume of sample used

### 2.5 The Experimental Design and Statistical Analysis

Response surface methodology (RSM) is a method for examining the connection between process factors and responses. As a result, the term reaction is often used to define a performance statistic or a quality aspect. The input variables, also known as independent variables, are under the control of the scientist or engineer [56]. The response‐surface approach is a set of methodologies for determining the ideal operating conditions using experimental approaches. Typically, this entails carrying out a succession of experiments and using the outcomes of one to drive the next [57]. In this experiment, 1.55 g/L of biochar was added to the stock solution constantly. These inputs, as indicated in Table 1, provide the number of experimental runs, current, pH range, duration, and electrolytes generated by the RSM program.

**TABLE 1:** Removal of Hg from Stock solution at 1 cm and 0.5 cm electrode distance by electrochemical oxidation process.

Factors					Responses			
Run	**A**: **pH**	**B**: **time** **(min)**	**C**: **Concentration** **(mg/L)**	**D, Current** **(amp)**	**Voltage** **(V)**	Hg Removal ED = 1 cm	**Hg** **Removal** **ED = 0.5 cm**	**Power consumed** **Kwh/m** ^**3**^
1	5	40	225	0.35	1.40	96.0401	97.5901	0.33
2	5	30	225	0.35	1.38	96.2381	97.7881	0.24
3	5	40	475	0.55	1.61	94.6771	96.2271	0.59
4	7	35	350	0.45	1.70	96.4991	98.0491	0.45
5	9	40	475	0.35	1.34	92.4881	94.0381	0.31
6	7	35	100	0.45	1.70	97.1401	98.6901	0.45
7	7	35	350	0.25	0.94	94.2621	95.8121	0.14
8	9	40	225	0.55	1.70	92.5171	94.0671	0.62
9	11	35	350	0.45	1.65	89.7151	91.2651	0.43
10	9	40	225	0.35	1.48	93.7861	95.3361	0.34
11	7	35	600	0.45	1.80	95.3321	96.8821	0.47
12	5	40	475	0.35	1.36	94.8901	96.4401	0.32
13	7	35	350	0.65	1.84	93.2541	94.8041	0.70
14	5	40	225	0.55	1.70	95.6871	97.2371	0.62
15	9	40	475	0.55	1.73	91.6531	93.2031	0.63
16	9	30	225	0.55	1.72	92.9831	94.5331	0.47
17	7	25	350	0.45	1.81	94.8851	96.4351	0.34
18	5	30	475	0.55	1.83	95.6791	97.2291	0.50
19	9	30	475	0.55	1.81	92.7321	94.2821	0.50
20	9	30	475	0.35	0.78	93.1441	94.6941	0.14
21	7	35	350	0.45	1.80	96.5991	98.1491	0.47
22	7	45	350	0.45	1.82	93.6291	95.1791	0.61
23	7	35	350	0.45	1.83	96.7031	98.2531	0.48
24	5	30	225	0.55	1.68	95.9361	97.4861	0.46
25	7	35	350	0.45	1.83	96.3691	97.9191	0.48
26	9	30	225	0.35	1.71	94.2761	95.8261	0.30
27	3	35	350	0.45	1.76	95.1041	96.6541	0.46
28	7	35	350	0.45	1.81	96.6091	98.1591	0.47
29	7	35	350	0.45	1.82	96.6791	98.2291	0.48
30	5	30	475	0.35	1.75	95.3161	96.8661	0.31

Using codes in experimental design, experimental factors are normalized to a defined scale, facilitating easier comparisons across components and simplifying statistical analysis. Coded low, which is usually denoted by −1 in the coded scale, is the bottom bound of a factor's range [52]. This makes the experiment's mathematical models simpler. Coded high, which is usually denoted by +1 in the coded scale, is the upper bound of a factor's range. With this coding, variables with varying scales and units such as the actual pH range of 5–9 can be interpreted and compared more easily [58].

### 2.6 Biochar Adsorbent

The biochar adsorption experiments were conducted in batch mode. A predetermined amount of biochar adsorbent was added to synthetic mercuric chloride wastewater prepared in 1L for each experimental run. The bottles were then placed on a magnetic stirrer for agitation during the combined adsorption and PEO process. The contact time between the biochar adsorbent and the wastewater was varied to determine the optimal duration for maximum mercury (II) ion removal. The initial concentration of mercury (II) ions in wastewater and the dosage of biochar adsorbent were varied to investigate their influence on the adsorption capacity. The initial concentration was adjusted by diluting the synthetic mercuric chloride wastewater with deionized water. The biochar dosage refers to the amount of biochar adsorbent added to the wastewater, usually expressed in grams per unit volume.

#### 2.6.1 Biochar Preparation

Biochar was produced from agricultural waste, specifically rice husk, using a pyrolysis process. The raw biomass feedstock was collected, air‐dried, and shredded into smaller particles. The shredded biomass was then pyrolyzed in a laboratory‐scale pyrolysis reactor under anoxic conditions at a temperature range of 500‐700°C. The pyrolysis process was carried out for 2–3 h to ensure the complete conversion of the biomass into biochar. Finally, 1.55 g/L biochar was added constantly for each experimental run. The physiochemical characteristics of biochar were analyzed using proximate analysis. The biochar had a moisture content of 7.07%, with a ash content of 5.22%, possibly due to residual inorganic compounds in the adsorbent. The volatile matter and fixed carbon were 21.26% and 66.45%, respectively. The high fixed carbon value suggests a high graphitization grade and low functional group count, suggesting that a lower AC value may be more effective for adsorption of contaminated wastewater.

## 3 Results and Discussion

### 3.1 Removal of Hg (II) from Wastewater at 0.5 and 1 cm Electrode Distance by PEO Process

To improve conductivity, 1 g of NaCl was added as well and 1.55 g of biochar adsorbent per 1 g/L Hg stock solution were added to the distilled water according to the prepared concentration of stock solution during calibration in this investigation. NaCl is an electrolyte that forms Na^+^ and Cl^−1^ during the removal of Hg (II) from synthetic wastewater using the PEO technique [59]. Diluted 0.02N H_2_SO_4_ and 0.02N NaOH were also employed in the procedure to achieve the necessary pH value. Al electrodes were employed in this Hg (II) removal experiment at 0.5 and 1 cm gap distances. Table **1** shows the removal efficiency summary of the method by using Al electrode with 0.5 and 1 cm distance.

### 3.2 Removal of Mercury (II) from Cement Production Wastewater Using PECO and Biochar Adsorption

Cement factories use large volumes of water for cooling, which can be contaminated with heavy metals, particulate matter, and other contaminants if not handled properly [60]. Water used in wet scrubbers and emission control devices can also be contaminated during combustion. Dust suppression and cleaning systems may also contain suspended particles, grease, and oil. Alkaline materials, trace levels of heavy metals, and tiny particulate matter can also pollute process water. Contaminants in cement industry wastewater include suspended solids, heavy metals, alkalinity, oil and grease, and sulfur compounds [61].

Using real wastewater that has mercury from the cement industry, the effectiveness of PEO combined with biochar as a material to absorb contaminants was tested and confirmed. The best removal rates were 97.37% and 96.24% with PEO alone, and 98.92% and 97.89% when PEO was used with magnetic biochar at 0.5 and 1 cm electrode spacing, respectively, along with paired electrooxidation. At pH 6.03, 34.15 min of reaction time, 1.55 g of magnetic biochar dosage, 0.43 amps of current, 0.45 kWh/m^3^ of electric energy consumed, and 227 mg/L of mercury concentration, these results were collected of energy usage needed to accomplish this. The present electric power utility cost in Ethiopia was $0.015/kWh.

### 3.3 Effect of Operating Parameters on % Removal of Mercury

#### 3.3.1 Effect of Electrode Distance

The study uses Al plates with 0.5 and 1 cm gap lengths between electrodes and considers factors like ED and mercury removal effectiveness in stock solutions [62]. There are several explanations why mercury removal effectiveness increases with smaller electrode spacing. First, greater interactions between mercury ions and electrodes are made possible by an increase in the electric field intensity between the electrodes [63]. Second, closer electrode spacing produces larger current densities, which speed up electrochemical processes and increase the effectiveness of mercury removal [64]. Third, shorter electrode lengths shorten the mercury ions’ diffusion route, facilitating faster mass transfer and more efficient mercury removal from the stock solution [65]. Fourth, a better distribution of electric potential between electrodes is made possible by decreased electrode spacing, which also lessens the effects of electrode polarization. To conclude, for effective mercury removal in a stock solution, it is critical to strike the correct balance between electrode distance and other operating factors.

#### 3.3.2 Effect of pH

**FIGURE 2:**
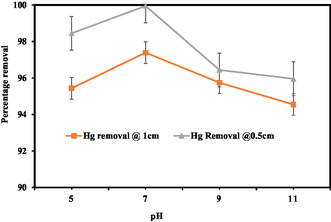
Effect of pH on removal efficiency by using PEO assisted by biochar adsorbent.

The degree to which a liquid is acidic or alkaline is referred to as its pH. Waters with higher alkalinity have a higher pH than those with lesser alkalinity. The pH of the solution is important for the elimination of pollutants in EC techniques. To evaluate the influence of pH on the efficiency of the electrocoagulation process for the removal of impurities, this study integrated pH ranges from 3 to 11 into the solution while maintaining constant values for concentration, contact duration, and current (0.43 A) and constant dose of 1.55 g/L of biochar adsorbent. From Figure 2, the value of pH discussed after treatment is based on the CCD‐designed result. the removal of Hg (II) at 1 cm ED increased from 95.563% to 97.899% when pH increased from 3 to 6, but when pH increased from 6 to 11, the removal declined from 89.89 to 91.47%. The maximum removal is achieved at pH 6.03. It seems more likely that the neutral conditions will reduce Mg removal. Due to a significant decline in the redox potentials of H^+1^ with lowering pH, which occurs when more oxidant is produced in the neutral medium, it decreases in the basic medium. Using aluminum electrodes, the best COD removals could be obtained at the neutral pH value [49, 51, 52].

Removal of Hg (II) at 1 cm ED increased from 95.86 to 97.12% when pH increased from 3 to 7, but when pH increased from 6 to 11, the removal declined from 97.12 to 89.47%, so the maximum removal efficiency achieved at pH 6.03 was 97.12%. Removal of mercury at 0.5 cm, ED increased from 97.69 to 98.12% when pH increased from 3 to 6.03, but when pH increased from 6 to 11, the removal decreased from 98.12 to 91.75%, so maximum removal efficiency was achieved at pH 6.03 with 98.92%. There are many reasons for the highest mercury removal effectiveness in PEO at pH 6.03. These include pH‐dependent interference, ideal reaction kinetics, electrode performance, and chemical speciation, which impact the reactivity and removal efficiency of mercury. Depending on the pH level, mercury can exist at pH 6.03 in a certain chemical form that is more prone to oxidation [66]. At this pH level, the particular electrode potential and reaction rates are most advantageous for the PECO process, which may display excellent reaction kinetics [62, 67] . Although these considerations are predicated on a broad understanding of electrochemical processes and pH effects, other compounds present in wastewater or solutions may potentially interfere with the mercury removal process.

#### 3.3.3 Effect of Initial Concentration

**FIGURE 3:**
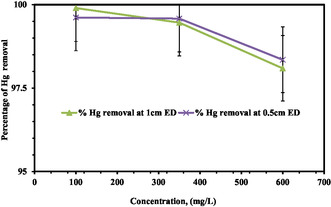
Effect of initial concentration on removal efficiency by using PEO supported by biochar adsorbent.

In this study, as shown in Figure 3, the concentration used in the process was arranged from 100 to 600 mg/L, and a constant dose of 1.55 g/L of biochar adsorbent while other parameters were constant. Concentration assesses the impact on the removal effectiveness of the PEO. From Figure 3, the value of concentration discussed after treatment based on the CCD‐designed result. Figure 3 shows that the removal of mercury at 1 cm ED decreased from 97.14 to 95.69% when the concentration increased from 100 to 600 mg/L. Removal of Hg (II) at 0.5 cm ED decreased from 98.69 to 96.88% when concentration increased from 100 to 600 mg/L and constant dose of 1.55 g/L of biochar adsorbent. So, the maximum removal efficiency optimized by RSM at 227 mg/L was 98.92%. The PEO is a method that extracts pollutants, such as mercury, from wastewater through electrical currents. Because the available electrical energy is used efficiently, removal efficiency is high when the content of mercury in wastewater is low [68]. The reason for this is that there are fewer mercury ions, which makes it possible for the electrical current to react and neutralize them [36].

Mercury ions (Hg^2+^) are mostly neutralized during PEO at the cathode by electrochemical reduction to elemental mercury (Hg^0^). Following reduction, the mercury may precipitate out of the solution, deposit on the electrode, or undergo additional oxidation to create insoluble mercury compounds [69]. Mercury ions in contaminated water or other solutions can be effectively neutralized with PECO because of the pulsed character of the current, which facilitates these processes [10]. During PEO, a combination of electrochemical reduction, oxidation, and perhaps precipitation processes can neutralize mercury ions (Hg^2+^) in a solution [15]. However, when the quantity of mercury is high, the available electrical energy is inadequate to oxidize all of the mercury ions present in the effluent, which results in a drop in removal efficiency [70].

#### 3.3.4 Effect of Reaction Time

**FIGURE 4:**
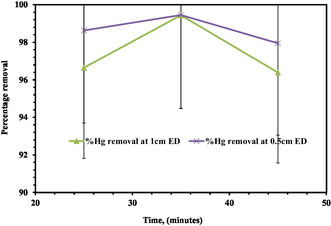
Effect of contact time PEO supported by biochar adsorbent on Hg removal efficiency.

The structure of the sludge may change over time, influencing the effectiveness of pollutant removal as well as the settling ability and floatability of the flocs. According to the study, very lengthy reaction periods result in reduced removal percentages, which might be attributed to metal hydroxide sequestration at the electrode level. In this experiment, the contact time ranges from 25 to 45 min. As a consequence of the wastewater treatment results, lengthy and very short times have low removal efficiency, as shown in Figure 4 below. According to the experimental protocol, the contact duration employed in this investigation ranged from 25 to 50 min, as shown in Figure 4. The impact of contact time on the removal efficacy of the PEO process is evaluated.

Removal of mercury at 1 cm ED increased from 96.24 to 97.89% when contact time increased from 25 to 35 min. But, when contact time increased from 34.15 to 45 min, the removal declined from 98.92 to 96.69%, so the maximum removal efficiency achieved at 34.15 min was 98.92%. The PEO is a method that converts mercury ions into insoluble compounds, which can be removed from wastewater. The effectiveness of this process depends on the amount of mercury present and the electrolysis duration. Low concentrations allow for more interaction between pulses, enhancing the efficiency of oxidation and removal [54]. The removal of mercury can be hindered by the length of the electrolysis period, as overexposure to electrical pulses can cause side reactions or by‐products [71]. The removal effectiveness of mercury may be reduced by short electrolysis periods that do not provide enough interaction between the mercury ions and the electrical pulses [54, 70].

#### 3.3.5 Effect of Current Density

**FIGURE 5:**
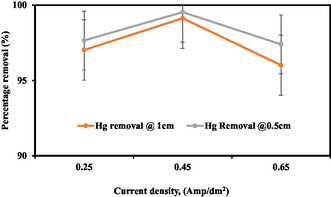
Effect of current density on removal efficiency by using the PEO.

In this study, the current density used in the process was arranged from 0.25 to 0.65 A according to the procedure of the experiment. Current density assesses the impact on the removal effectiveness of the PEO. From Figure 5, the value of the current density discussed after wastewater treatment

Removal of Hg at 1 cm ED increased from 97.02 to 98.26% when the current density increased from 0.25 to 0.43 A. But, when the current density increased from 0.45 to 0.65 A, the removal declined from 98.26 to 96.01%, so the maximum removal efficiency achieved at 0.45 A was 98.26%. Removal of Hg at 0.5 cm ED increased from 96.12 to 98.81% when the current density increased from 0.25 to 0.65 A. But, when the current density increased from 0.43 to 0.65 A, the removal declined from 98.92 to 98.81%, so the maximum removal efficiency achieved at 0.43 A was 99.03%. In a process known as PEO, the applied current or voltage affects the removal efficiency. Because low current/voltage restricts the formation of reactive species and their capacity to break down target contaminants, it causes sluggish reaction kinetics and decreased efficiency. On the other hand, high current/voltage can cause electrode passivation and unintended side reactions but also speed up reaction kinetics and encourage the production of reactive species [51]. To guarantee adequate reaction kinetics and the production of reactive species while reducing side reactions and electrode the passivation process moderate current/voltage values are recommended. The optimal current/voltage range for good removal efficiency should also consider the unique properties of the target contaminants and electrode material [68].

### 3.4 Comparisons with Other Existing Technology

The environment is seriously threatened by inorganic contaminants, especially heavy metals. Harmful and carcinogenic, these heavy metals are highly dangerous substances [72]. Anodic redissolution in the differential pulse mode was used to analyze the removal from the treatment. When the distance between the electrodes was 3 cm and the current density ranged from 2.5 to 3.125 Adm^−2^, the removal efficiency was above 99.9%; for example, 99.95% of the mercury (II) was eliminated when charge loadings of 9.33 and 15.55 Fm^−3^ were applied for Fe and Al, respectively. Under these circumstances, changing the mercury (II) solutions’ pH from 3 to 7 maintained a clearance effectiveness of greater than 99% [72], by iron oxide nanoparticles (IOPN) mercury removal of 70% approximately was confirmed by atomic absorption spectroscopy measurements [73]. Our study's total mercury removal levels from chloralkaline wastewater ranged from 90 to 98%, demonstrating the excellent efficacy of the microbial de‐toxification system for mercury in situ wastewater settings [69]. A maximum removal percentage of around 87% was noted from an initial Hg (II) concentration of 8 mg/L; the removal percentages (%) for 6 mg/L and 4 mg/L are ≈73% and 68%, respectively. The removal percentages (%) at 10 mg/L and 25 mg/L decreased as the initial Hg (II) concentration increased. This is because the iron oxide nanoparticles (IOPN) sites eventually became saturated with adsorbed Hg (II), at which point adding more Hg (II) to the solution would hardly increase the amount of adsorbed Hg (II). The elimination percentage (%) at 50 mg/L was less than 50% [73]. Adsorption is one of the many technologies that have been created over the years for the removal of mercury, and it is a method that works well for purifying water since it is easy to use, relatively inexpensive, and simple. Mercury was extracted using a thiol‐rich 3D‐porous hyper cross‐link polymer, with up to 98% removal efficiency [70]. In comparison to values observed for sanitary determinants (84.7–99.9%), the overall removal effectiveness for mercury (66.3%) was low. Significantly less mercury was removed from the system during the secondary treatment stage (29.5%) than during the primary treatment stage (52.2%) [10]. The study found that optimal conditions for decolorization include pH = 6, 300 mA electrical current, 3 cm distance between anodes, and 40 min reaction time. These conditions achieved 98% color and 87% COD removal, with rate constants in agreement with the 1^st^‐order kinetic model. Pulsed electro‐oxidation was the most effective in 1 h, reducing total organic carbon (TOC), chemical oxygen demand (COD), and biological oxygen demand (BOD) [74, 75].

### 3.5 Analysis of Variance Test

Analysis of variance (ANOVA) was used to evaluate the statistical significance of the quadratic model. The significance of the model and the model terms are decided by using the F‐test and p‐test. The higher the magnitude of F and the smaller the *p*‐value, the more significant the conforming model and model term s [76, 77]. The correlation coefficient (R^2^) value was used to evaluate the fit of the polynomial model, while the F test was used to ascertain its statistical significance. The *p* value (probability) was used to evaluate model terms with a 95% degree of confidence. ANOVA is a statistical technique used to test hypotheses about a model's parameters by dividing the total variance into smaller groups and component portions linked to specific sources of variation. The experiments were conducted at random to avoid systemic errors. The performance of independent variables is determined by the coefficients of the 2^nd^‐order model, which interprets the amount of removal of researched parameters. The ANOVA findings for responses with probability values *p* < 0.0500 indicate the significance of the 2^nd^‐order model [58, 78]

#### 3.5.1 ANOVA for the % of Hg Removal Using PEO @1 Cm Electrode Distance by Quadratic Model

As shown in Table 2, the model F‐value of 458.57 implies the model is significant. There is only a 0.01% chance that an F‐value this large could occur due to noise. *p*‐values less than 0.05 indicate model terms are significant. In this case A, B, C, D, AD, BC, CD, A^2^, B^2^, C^2^, D^2^ are significant model terms. Values greater than 0.10 indicate the model terms are not significant. If there are many insignificant model terms (not counting those required to support hierarchy), model reduction may improve model. The lack of fit F‐value of 0.91 implies the lack of fit is not significant relative to the pure error. There is a 58.19% chance that a lack of fit F‐value this large could occur due to noise.

**TABLE 2:** ANOVA for the percentage removal of Hg at 1 cm Electrode distance (ED) by the quadratic model using PECO.

Source	Sum of Squares	df	Mean Square	F‐value	*p*‐Value
**Model**	93.05	14	6.65	458.57	<0.0001 Significant
A‐pH	41.77	1	41.77	2881.90	<0.0001 Significant
B‐Contact time	2.09	1	2.09	144.02	<0.0001 Significant
C‐Concentration	4.59	1	4.59	316.94	<0.0001 Significant
D‐Current	1.67	1	1.67	115.19	<0.0001 Significant
AB	0.0416	1	0.0416	2.87	0.1108
AC	0.0027	1	0.0027	0.1830	0.6749
AD	0.6823	1	0.6823	47.07	<0.0001 Significant
BC	0.1936	1	0.1936	13.36	0.0023 Significant
BD	0.0658	1	0.0658	4.54	0.0501
CD	0.2809	1	0.2809	19.38	0.0005 Significant
A^2^	28.94	1	28.94	1996.47	<0.0001 Significant
B^2^	8.76	1	8.76	604.64	<0.0001 Significant
C^2^	0.1363	1	0.1363	9.41	0.0078 Significant
D^2^	13.06	1	13.06	900.98	<0.0001 Significant
**Residual**	0.2174	15	0.0145		
Lack of Fit	0.1403	10	0.0140	0.9093	0.5819
Pure Error	0.0771	5	0.0154		
**Cor Total**	93.27	29			

#### 3.5.2 ANOVA for the % of Hg Removal Using PEO @0.5 Cm Electrode Distance by Quadratic Model

Table 3 shows that the model F‐value of 467.57 implies the model is significant. There is only a 0.01% chance that an F‐value this large could occur due to noise. *p*‐values less than 0.0500 indicate model terms are significant. In this case A, B, C, D, AD, BC, CD, A^2^, B^2^, C^2^, D^2^ are significant model terms. Values greater than 0.10 indicate the model terms are not significant. If there are many insignificant model terms (not counting those required to support hierarchy), model reduction may improve your model. The lack of fit F‐value of 0.93 implies the lack of fit is not significant relative to the pure error. There is a 58.62% chance that a lack of fit F‐value this large could occur due to noise. The lack of fit F‐value of 0.90 implies the lack of fit is not significant relative to the pure error [58].

**TABLE 3:** ANOVA for the percentage removal of Hg at 0.5 cm ED by quadratic model.

Source	Sum of Squares	df	Mean Square	F‐value	*p*‐Value
**Model**	93.05	14	6.65	458.57	<0.0001 Significant
A‐pH	41.77	1	41.77	2881.90	<0.0001 Significant
B‐Contact time	2.09	1	2.09	144.02	<0.0001 Significant
C‐Concentration	4.59	1	4.59	316.94	<0.0001 Significant
D‐Current	1.67	1	1.67	115.19	<0.0001 Significant
AB	0.0416	1	0.0416	2.87	0.1108
AC	0.0027	1	0.0027	0.1830	0.6749
AD	0.6823	1	0.6823	47.07	<0.0001 Significant
BC	0.1936	1	0.1936	13.36	0.0023 Significant
BD	0.0658	1	0.0658	4.54	0.0501
CD	0.2809	1	0.2809	19.38	0.0005 Significant
A^2^	28.94	1	28.94	1996.47	<0.0001 Significant
B^2^	8.76	1	8.76	604.64	<0.0001 Significant
C^2^	0.1363	1	0.1363	9.41	0.0078 Significant
D^2^	13.06	1	13.06	900.98	<0.0001 Significant
**Residual**	0.2174	15	0.0145		
Lack of Fit	0.1403	10	0.0140	0.9093	0.5919
Pure Error	0.0771	5	0.0154		
**Cor Total**	93.27	29			

### 3.6 Fit Statistics

The study utilized ANOVA to analyze the statistical significance of a model equation and terms, controlling the model fitting quality with determination coefficients (R^2^ and Adj.R^2^) and the Fischer test for statistical significance (F‐test) [49]. The model was validated using predicted R^2^ and adjusted R^2^ to evaluate its prediction power. The model's goodness‐of‐fit was assessed using coefficients of determination R^2^ (correlation coefficient) and adjusted coefficients of determination adj R^2^. The high correlation coefficient (R^2^ = 0.99) and the model's ability to account for response variability demonstrated its excellent dependability in forecasting elimination percentages [58]. Table 4 shows that the predicted R^2^ of 0.99 is in reasonable agreement with the adjusted R^2^ of 0.99; i.e., the difference is less than 0.2. Adeq precision measures the signal to noise ratio. A ratio greater than 4 is desirable. The ratio of 86.22 indicates an adequate signal. This model can be used to navigate the design space. As shown in Table 5, the predicted R^2^ of 0.9974 is in reasonable agreement with the Adjusted R^2^ of 0.99;, i.e., the difference is less than 0.2. Adeq precision measures the signal‐to‐noise ratio. A ratio greater than 4 is desirable ratio of 85.430 indicates an adequate signal.

**TABLE 4:** Model summary for percentage %Hg removals at 1 cm ED by using PEO.

Std. Dev.	0.1204	R^2^	0.9977
Mean	94.69	Adjusted R^2^	0.9955
C.V. %	0.1271	Predicted R^2^	0.9901
		Adeq Precision	86.2219

**TABLE 5:** Model summary for percentage % Hg removals at 0.5 cm ED by using PEO.

Std. Dev.	0.1204	R^2^	0.9977
Mean	96.24	Adjusted R^2^	0.9955
C.V. %	0.1251	Predicted R^2^	0.9974
		Adeq Precision	85.2219

### 3.7 Effects of Model Parameters and Their Interactions

3D surfaces and 2D contour plots are useful for revealing reaction system conditions and analyzing the impact of variables. These graphical representations of regression equations help visualize the response functions of two elements within experimental ranges. The results show that all combined process variables have a significant effect on pollution removal efficiency in synthetic wastewater. The effect of interaction indicates that more than two independent variables can affect the efficiency of pollution removal.

#### 3.7.1 Interaction of Time, Concentration, and Current

**FIGURE 6:**
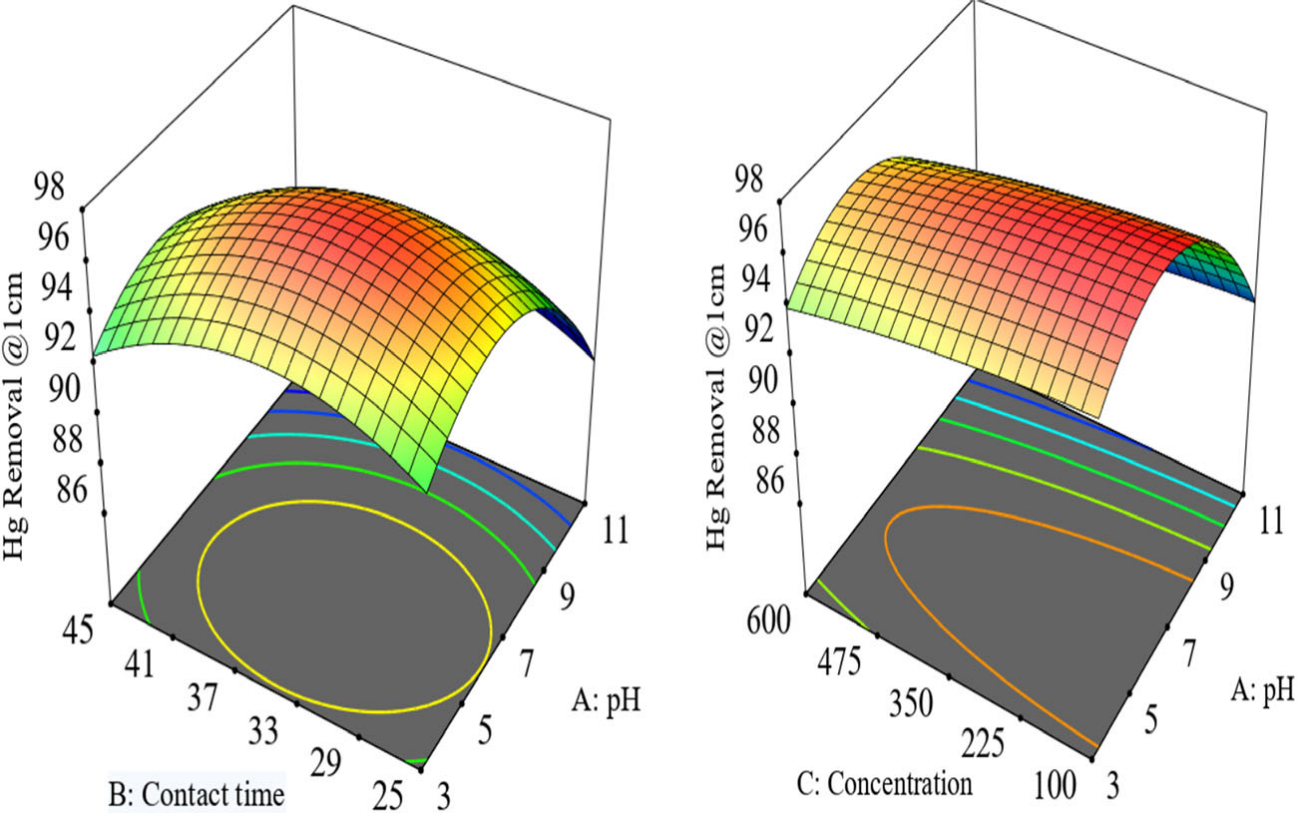
Interaction effect of time, pH and concentration with for % removal at 1 cm electrode distance on removal of mercury from stock solution.

**FIGURE 7:**
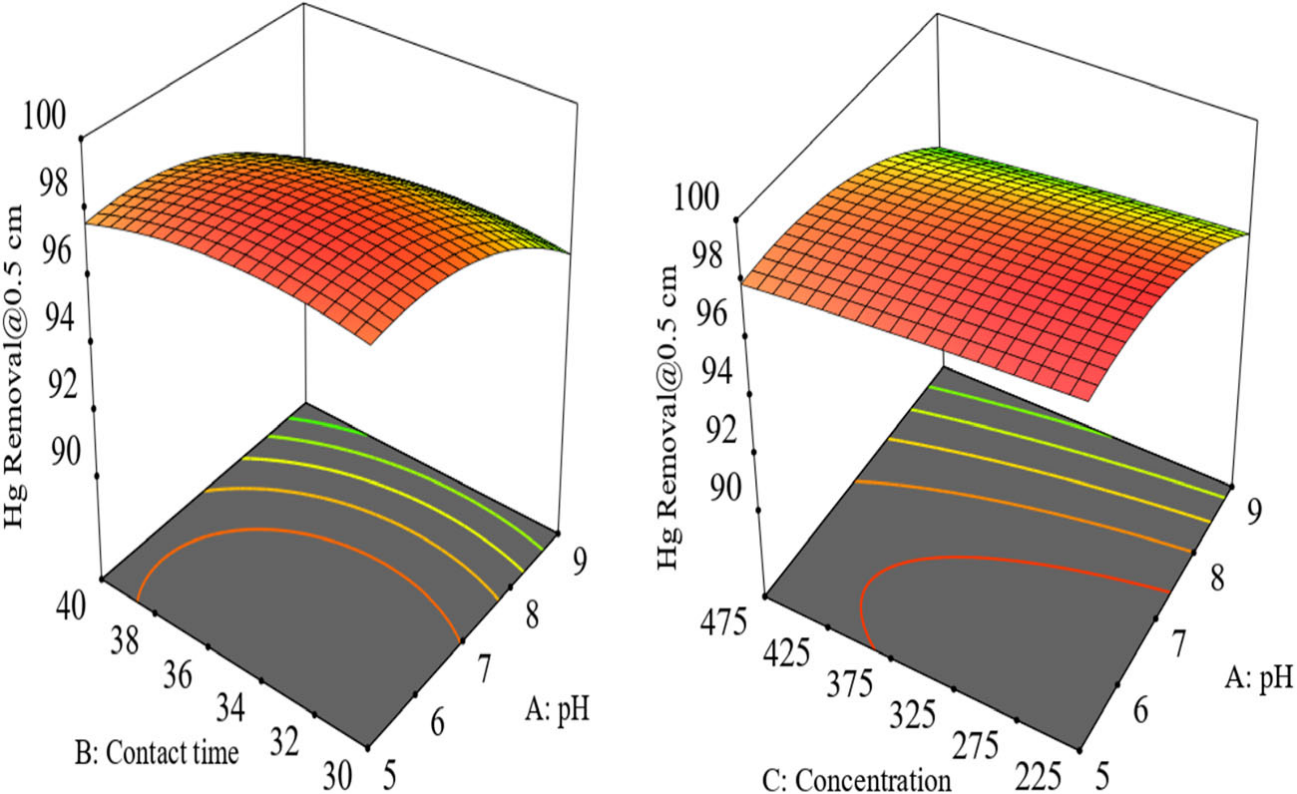
Interaction effect of contact time with pH, time, and concentration plots for % removal of mercury from the stock solution at 0.5 cm electrode distance.

The 3D figure demonstrates high Hg removal efficiency at 0.45 amp and 35 min. The graph shows that increasing the time and current from 0.25 amp to 0.45 amp leads to higher efficiency. Green points indicate lower efficiency, and the interaction effect of current and time was significant in the electrooxidation process at 1 cm ED, aluminum (Al–Al), as shown in figure 6 and 7.

#### 3.7.2 Interaction of Contact Time, Concentration, and pH

The 3D Figures 6 and 7 illustrate the relationship between mercury (Hg) removal efficiency, pH, and contact duration. It shows that higher pH values around 7, highlighted in red, and contact times of ≈34.15 min result in greater Hg removal percentages (97–98%). The graph reveals that the optimal conditions for Hg removal, achieving around 98.92% efficiency, are found at pH levels between 9 and 11 and contact times ranging from 25 to 45 min. Neutral pH values correspond to higher removal efficiencies, emphasizing the significant role pH plays in the process. While very long contact times lead to lower removal percentages, the maximum efficiency occurs at a medium duration of around 34 min, as indicated by the red color. Overall, the graph suggests that both pH and contact duration should be optimized to achieve the highest possible Hg removal efficiency [79, 80]

### 3.8 Regression Equations

Regression analysis is a statistical method used to determine the relationship between a dependent variable and predictor variables [81]. It helps in determining the correctness of outcomes and the dualities of the fit. The final model's optimal values were obtained using numerical methods. The experiment examined pH, initial concentration, reaction time, and current density to find optimal operating parameters for maximum removal efficiency of Hg. These elements were tuned during the pulsed EO procedure to achieve excellent removal efficiency. An empirical association between the response and independent variables for electrocoagulation was discovered [82, 83]

**% Removal of Hg at 1 Cm ED**

(16) Hg removal at 1 cm=96.58‐1.32A‐0.294B‐0.435C‐0.2638D‐0.051AB+0.0143AC‐0.5371AD‐0.341BC‐0.107BD+0.126CD

**% Removal of Hg at 0.5 Cm ED**

(17) Hg removal at 0.5 cm=98.999‐0.926A+0.125B‐0.204C+0.148D‐0.017AB‐0.136AC‐0.102AD‐0.214BC+0.103BD‐0.172CD

The study used a coded equation to predict the response of various factors in wastewater treatment. High levels were coded as + 1, while low levels were coded as −1. The equation identified the relative impact of these factors by comparing their coefficients. The model terms included pH, initial concentration, reaction time, current density, and interaction terms. The ANOVA test was used to determine the model's appropriateness, while the lack of fit test confirmed its validity. The regression model showed a significant difference (*p* < 0.05), indicating that EC was the most effective method for wastewater treatment due to the higher percentage of Hg. Significant differences (*p* ≤ 0.05) were shown by means with different letters in the same row.

### 3.9 The Condition for Responses Desired Optimum

The study found that the prediction and experimental findings were in line with a straight line and good agreement, indicating the model's validity. The initial model's expected R^2^ was 99.09%, and the backward elimination method was applied to build a parsimonious model with meaningful predictors. The anticipated model's coefficient of determination revealed a quadratic link between responses and parameters with a good regression coefficient. The optimum PEO process was obtained using Design Expert 13 software and tested under optimal conditions. The experimental results for removal efficiencies were very close to the predicted values, with a difference of less than 0.2 and no significant differences (*p* > 0.05) [33].

**FIGURE 8:**
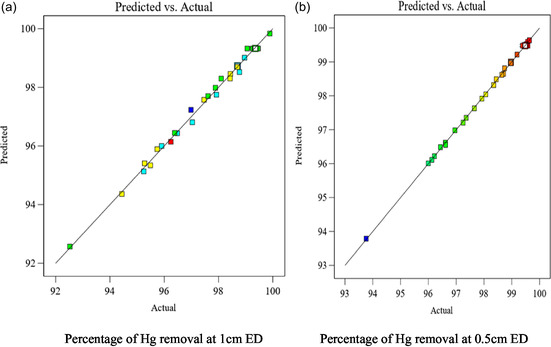
(a) Percentage of Hg removal at 1 cm ED and (b) Percentage of Hg removal at 0.5 cm ED: Comparison of the predictive and experimental results for pulsed electrochemical oxidation supported by biochar on removal efficiency.

The models accurately forecast the removal of Hg and other contaminants, as shown in Figure 8. The interactive link between four independent factors and dependent variables can be visualized using regression models and aligned diagrams. (a), indicating that all the data points were distributed along the 45^o^ line, A normal probability plot indicates that the residuals follow a normal distribution, and the points follow a straight line. used in judging whether the models are satisfactory.

The figure indicated that the data were plotted against a theoretical normal distribution, and the points should form an approximately straight line and a departure from this line would point out the departure from a normal distribution. From this graphical result, the data points were very small deviating from the normal distribution given, but not very critical. Diagnostic diagrams, such as the predicted values versus real values, and the normal probability distribution diagram of residuals can assess the model's suitability.

**FIGURE 9:**
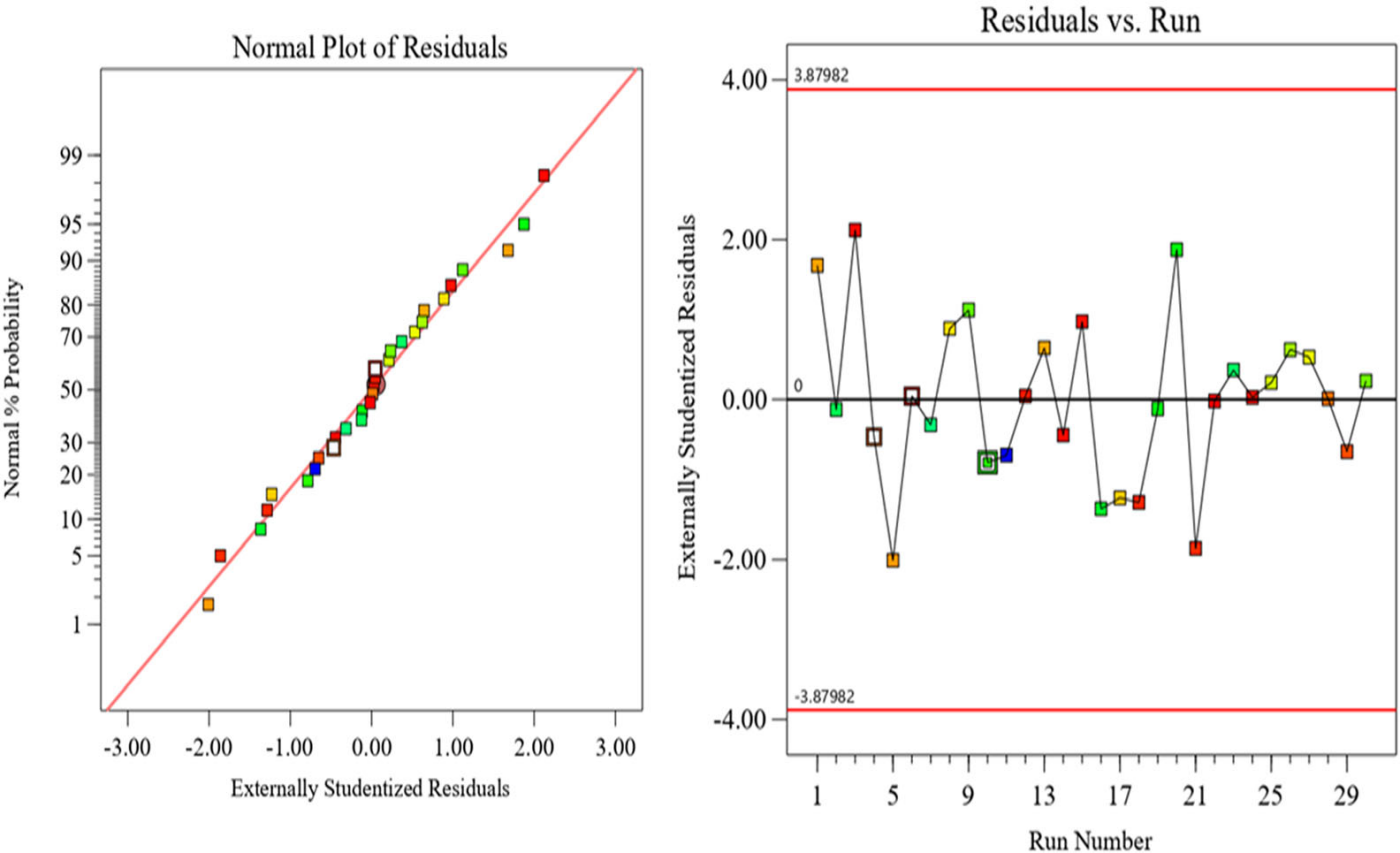
Distribution of normal probability % and residuals for Hg at 1 cm ED.

**FIGURE 10:**
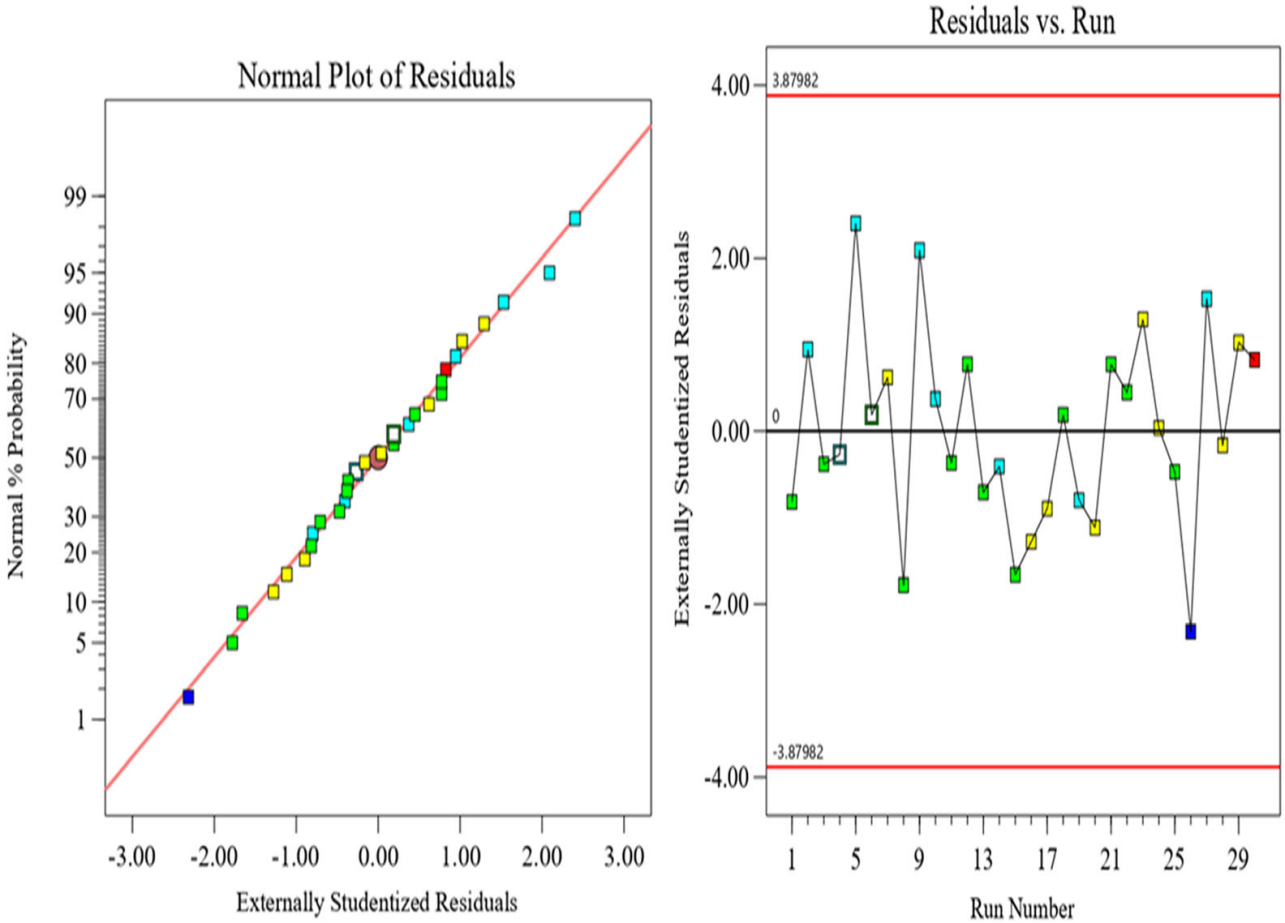
Distribution of normal probability % and residuals for Hg at 0.5 cm ED.

Figures 9 and 10 show studentized residuals for Hg and the distribution of the normal probability percentage. As shown in the Figures 9 and 10 results of the removal efficiency was nearer to a straight line on the distribution of normal probability percentage and residuals graphs for pollutant removals. On the second graph, run numbers versus externally studentized residuals were in uniform variation. As a result, the experimental results were valid [52] and residuals can assess the model's suitability. Figures 9 and 10 show studentized residuals for Hg and the distribution of the normal probability percentage. As shown in Figures 9 and 10, the results of removal efficiency were nearer to a straight line on the distribution of normal probability percentage and residuals graphs for pollutant removals. On the second graph, run numbers versus externally studentized residuals were in uniform variation. As a result, the experimental results were valid [52].

### 3.10 Energy Consumption

The PEO‐Biochar system was able to treat biochar with a specific energy consumption of 0.45 kWh/m^3^ under optimized conditions, which is equivalent to or more efficient than most conventional methods used for the removal of mercury from various water sources and effluents, such as ion exchange (0.5–2.0 kWh/m^3^ including pumping and resin regeneration) and chemical precipitation methods (0.2 to 0.8 kWh/m^3^ for mixing & pH adjustment plus additional energy & cost associated with chemical production. Additionally, the use of pulsed electrocoagulation processes has been reported to utilize greater amounts of energy than the pulsed PEO‐biochar process (e.g., 0.8–3.5 kWh/m^3^ depending on current density and operational parameters), which indicates that the pulsed mode of operation used to treat biochar will provide greater efficiency through decreased passivation of the electrode and improve mass transfer resulting in reduced electrical energy consumption while demonstrating that mercury removal rates exceed 98%, thus supporting the conclusion that PEO‐biochar process is substantially energy efficient and operationally competitive with existing mercury treatment technologies; therefore, this study provides empirical evidence to support the development of commercially viable PEO‐biochar system.

### 3.11 Economic Feasibility

Under optimized conditions, the energy consumption of the process was 0.45 kWh/m^3^, which corresponds to an estimated electricity cost of approximately $0.00675 per m^3^ based on the current Ethiopian electricity tariff of $0.015/kWh, indicating low operational energy costs. The system employs Al electrodes that act as sacrificial anodes during operation; although electrode dissolution contributes to material replacement costs, the PEO mode reduces electrode passivation, which can enhance electrode lifespan and lower maintenance frequency compared to continuous systems. Additionally, the biochar used was produced from rice husk via pyrolysis and applied at 1.55 g/L, suggesting that material costs can be minimized using low‐cost agricultural waste feedstocks, with further potential cost reduction through regeneration and reuse strategies. The combined PEO–biochar system also reduces electrode fouling and operational load, which may decrease long‐term operational expenses. Overall, the low specific energy consumption, inexpensive electrode material, availability of biomass‐derived adsorbent, and modular reactor configuration support the economic feasibility of scaling up, although future work will include a detailed techno‐economic assessment considering electrode consumption rate, biochar regeneration cycles, and sludge management under continuous real wastewater conditions

## 4 Conclusion

This study explored the use of PEO and biochar adsorption for the removal of mercury (II) from synthetic wastewater. The results showed that the method effectively promoted the removal of contaminants, with significant removal efficiencies achieved through specific parameters. The results indicated optimal removal efficiencies of 97.37% and 96.24% with PEO alone and 98.92% and 97.89% when PEO was combined with magnetic biochar for 0.5 cm and 1 cm electrode spacing, at pH 6.03, 34.15 min of reaction time, 1.55 g/L of magnetic biochar, 0.43 amps of current, and 0.45 Kwh/m^3^ of electric energy consumed*.* The biochar adsorbent demonstrated remarkable adsorption capacity for mercury ions, with its favorable surface properties facilitating strong interactions with target contaminants. The combined approach of PEO and biochar adsorption resulted in enhanced removal efficiencies compared to individual treatment methods. The study's significance lies in its potential application in real‐world wastewater treatment scenarios, offering a sustainable and environmentally friendly approach. However, the study's focus on synthetic wastewater and potential variations in performance under diverse wastewater conditions necessitate further research to evaluate the feasibility and effectiveness of the proposed approach. Future research should focus on optimizing operational parameters and ensuring the long‐term performance and stability of the biochar adsorbent for large‐scale applications.

## Author Contributions

**Werkne Sorsa Muleta**: data curation, resources, writing – original draft. **Lelisa Regea Mengistu**: conceptualization, methodology, validation, supervision. **Madhappan Santhamoorthy**: conceptualization, methodology, validation, supervision. **Perumal Asaithambi**: data curation, resources, writing – original draft.

## Conflicts of Interest

The authors declare no conflicts of interest.

## Data Availability

The data that support the findings of this study are available from the corresponding author upon reasonable request.
